# The Mechanosensitive Ion Channel Piezo1 Promotes Obliterative Bronchiolitis through YAP-dependent Epithelial-to-mesenchymal Transition

**DOI:** 10.2174/0115665240377965250701114255

**Published:** 2025-07-07

**Authors:** Li Wan, You Wu, Jinsong Yang, Peng Deng, Zuhuan Yao, Quanchao Sun

**Affiliations:** 1 Department of Thoracic Surgery, Union Hospital, Tongji Medical College, Huazhong University of Science and Technology, Wuhan, China; 2 Department of Gastrointestinal Surgery, Union Hospital, Tongji Medical College, Huazhong University of Science and Technology, Wuhan, China; 3 Cancer Center, Union Hospital, Tongji Medical College, Huazhong University of Science and Technology, Wuhan, China; 4 Division of Cardiothoracic and Vascular Surgery, Tongji Hospital, Tongji Medical College, Huazhong University of Science and Technology, Wuhan, China

**Keywords:** Piezo1, Yes-associated protein (YAP), epithelial-to-mesenchymal transition, obliterative bronchiolitis, lung transplantation, fibrosis

## Abstract

**Introduction:**

Obliterative bronchiolitis (OB) is a severe and progressive complication characterized by the fibrotic obliteration of small airways, leading to significant morbidity and mortality, particularly in lung transplant recipients. The pathogenesis of OB involves complex cellular processes, among which epithelial-to-mesenchymal transition (EMT) plays a crucial role. This study investigates the role of mechanosensitive ion channel Piezo1 in promoting OB through Yes-associated protein (YAP)-dependent EMT.

**Method:**

Piezo1-induced signal pathway alterations, fibrosis, and EMT-related features were examined in the mouse OB model and BEAS-2B cells. The efficacy of Piezo1 in EMT and OB was explored and validated both *in vitro* and *in vivo* .

**Results:**

Piezo1 was found to be upregulated in OB, and pharmacological inhibition of Piezo1 effectively alleviated EMT and fibrotic deposition. Piezo1 activation stimulated the Ca^2+^ influx and nuclear translocation of YAP that triggered the transition of epithelial cells into a mesenchymal phenotype, which contributed to airway fibrosis and obstruction. Furthermore, inhibition of YAP or calcium chelation significantly attenuated Piezo1 activation-induced EMT and OB, indicating that YAP and Ca^2+^ are critical mediators in this process.

**Discussion:**

Piezo1 expression was found to be upregulated in OB, and its activation induced the epithelial-to-mesenchymal transition (EMT) process *via* a YAP-dependent pathway. Piezo1 could accelerate EMT and the occlusion rate of grafts *via* Ca^2+^ influx-dependent YAP activation in OB, suggesting a direct role in facilitating EMT and subsequent fibrotic remodeling in OB.

**Conclusion:**

The present results highlight that Piezo1 promotes OB through a YAP-dependent EMT pathway, suggesting Piezo1 as a novel therapeutic strategy for treating OB and potentially improving outcomes of lung transplant recipients.

## 1 INTRODUCTION

Obliterative bronchiolitis (OB), a devastating complication of chronic lung allograft rejection, remains a significant barrier to long-term survival after lung transplantation [1]. Characterized by an insidious onset of airflow obstruction, OB is histologically defined by fibrotic occlusion of the small terminal airways and often linked to lung transplantation, hematopoietic stem cell transplants, and connective tissue diseases [2]. The pathogenesis of OB is complex and multifactorial, involving an interplay of immune responses, inflam-mation, airway epithelial injury, oxidative stress and hypoxia, and fibrotic processes [3, 4]. The incidence of OB post-transplantation has not significantly decreased despite improvement in immunosuppressive therapies, highlighting the need for a deeper understanding of its pathophysiological mechanisms [5, 6]. Epithelial-to-mesenchymal transition (EMT) is a complex biological process in which epithelial cells lose their polarity and adhesion properties, acquiring mesenchymal characteristics that contribute to fibrosis [7, 8]. Recent studies have implicated EMT as a key player in the fibrotic remodeling observed in OB [9]. EMT enables polarized epithelial cells to adopt migratory and invasive mesenchymal phenotypes through the suppression of adhesion molecules like E-cadherin and upregulation of mesenchymal markers, such as vimentin and N-cadherin [10]. This transition is orchestrated by a network of signaling pathways and molecular interactions, which, if deciphered, could unveil novel therapeutic targets [11].

Piezo1 is a mechanosensitive ion channel that converts mechanical stimuli into biochemical signals, which has garnered significant attention due to its pivotal role in various physiological functions, including cell proliferation, differentiation, immunity, and inflammation [12-15]. The lung is constantly subjected to mechanical forces due to respiration, which involves the rhythmic expansion and contraction of lung tissues [16-18]. Piezo1 is expressed in various cells of the lung, including epithelial cells, endothelial cells, and fibroblasts, and responds to these mechanical changes by allowing the influx of calcium ions. Calcium signaling is crucial for processes, such as maintaining barrier function, regulating inflammation, and tissue repair after lung injury [19, 20]. In the context of lung physiology and pathology, the role of Piezo1 has been increasingly recognized, with studies suggesting its involvement in pulmonary hypertension and lung edema [21]. However, the contribution of Piezo1 to the OB development has not yet been reported.

Yes-associated protein (YAP), a transcriptional co-activator, is a key component of the Hippo signaling pathway, which plays a critical role in regulating cell proliferation, differentiation, and survival during the pathological processes of many diseases [22, 23]. The dynamic interplay between YAP and EMT, a fundamental cellular reprogramming process, has emerged as a critical axis in developmental biology, cancer progression, and the development of fibrotic diseases. Recent studies have demonstrated that YAP activation can induce EMT in epithelial cells, leading to increased cell migration and invasion, which are hallmarks of metastatic cancer [24]. Similarly, in fibrosis, YAP activation in epithelial cells or fibroblasts exacerbates ECM deposition and tissue remodeling. YAP contributes to EMT by interacting with various transcription factors and signaling pathways, including TGF-β, Wnt/β-catenin, and Notch. These interactions facilitate the transcription of EMT-related genes, promoting the downregulation of epithelial markers (such as E-cadherin) and the upregulation of mesenchymal markers (such as α-SMA, N-cadherin, and vimentin) [25-27].

This study aimed to investigate the role of Piezo1 in the EMT process involved in the pathogenesis of OB, indicating its potential as a novel therapeutic target. We hypothesized that Piezo1 activation promotes EMT in lung epithelial cells, contributing to the fibrotic characteristics of OB. This study also enhanced the understanding of mechanical stress in OB progression after lung transplantation, offering insights into how mechanotransduction signals drive fibrosis and luminal occlusion.

## 2 MATERIALS AND METHODS

### 2.1 Animal Model and Experimental Design

This study utilized 28-32 female C57BL/6 and BALB/c mice, aged 8-12 weeks and weighing 28-32 g, obtained from Shulaibao Co., Ltd. (Wuhan, China). All of the animals were kept in a humidity-controlled and temperature-controlled (22±2℃) room facility (Tongji Medical College, Huazhong University of Science and Technology, Wuhan, People’s Republic of China) and fed with fresh water and standard laboratory food. All animal procedures were approved by the Institutional Animal Care and Use Committee (IACUC) and conducted in accordance with the National Institutes of Health guidelines for the care and use of laboratory animals.

Mice were randomly assigned into the following groups: control, isograft, isograft with a Piezo1 activator (Yoda1), isograft with a Piezo1 inhibitor (GsMTx4), allograft, allograft with Yoda1, and allograft with GsMTx4. OB was induced by orthotopic tracheal transplantation using donor mice as previously described [28]. Briefly, the donor mice were euthanized with pentobarbital sodium (80 mg/kg, i.p.) and the tracheas were carefully dissected under a microscope. A cervical midline incision was made to remove six cartilage rings starting from the second cartilage ring. After that, the grafts were cleaned, dried, and kept on ice with moist gauze covering them. The trachea was exposed *via* a cervical midline incision, which was made at the second cartilage ring beneath the cricoid cartilage. Subsequently, an 11–0 nonabsorbable suture was used to implant the tracheal grafts end-to-end in the recipients. To modulate Piezo1 activity, the Piezo1 antagonist GsMTx4 (1.0mg/kg, intraperitoneally) was applied daily starting from the first day after surgery [29]. The specific Piezo1 agonist Yoda1 (3 mg/kg) was administered intraperitoneally every other day post-surgery to activate the channel [30]. The control groups received an equivalent volume of saline.

### 2.2 Histological Analysis

Tracheas were harvested, fixed in 4% paraformaldehyde, and then embedded in paraffin, after which they were sectioned to a thickness of 4 µm. The slices were stained with Hematoxylin and Eosin (H&E) and Masson's trichrome staining for morphological observation and collagen deposition. The percentage of luminal occlusion was defined as follows: [(Area within the cartilage –Area of residual tracheal lumen) / Area within the cartilage] ×100% [28]. All the histological data were analyzed using Fiji software.

### 2.3 Immunofluorescence Staining

Immunofluorescence staining was performed to detect Piezo1 (1:100; MA5-32876, Invitrogen), E-cadherin (1:100; ab231303, Abcam), α-SMA (1:200; 701457, Invitrogen) and vimentin (1:100; ab92547, Abcam) expression in tracheal sections. After antigen retrieval by heating in citrate buffer, sections were incubated overnight at 4 °C with primary antibodies (listed below), followed by incubation with the appropriate secondary antibodies. The intensity of staining was scanned using a pathological section scanner (Leica SDPTOP HS6) and quantified using image analysis software.

### 2.4 Western Blot

The total protein was extracted from tracheal tissues or cultured cells, and the concentration was determined by the BCA assay. Equal amounts of protein were separated by SDS-PAGE and transferred to PVDF membranes. Membranes were blocked with 5% bovine serum albumin at room temperature for 1 h and incubated with primary antibodies. After incubation with HRP-conjugated secondary antibodies, protein bands were visualized using enhanced chemiluminescence and quantified by densitometry. The primary antibodies used in our study included anti-Piezo1 antibody (1:500; MA5-32876, Invitrogen), anti-E-cadherin antibody (1:1000; ab231303, Abcam), anti-α-SMA antibody (1:1000; 701457, Invitrogen), anti-vimentin antibody (1:1000; ab92547, Abcam), anti-YAP antibody (1:1000; ab205270, Abcam), anti-YAP phospho-S127 antibody (1:1000; ab76252, Abcam), anti-GAPDH antibody (1:1000; MA5-15738, Invitrogen), and anti-Histone H3 antibody (1:1000; ab1791, Abcam).

### 2.5 Quantitative PCR (qPCR)

Total RNA was extracted from tracheal tissues using TRIzol reagent, and cDNA was synthesized using a reverse transcription kit. qPCR was performed using SYBR Green on a real-time PCR system. The following primers for Piezo1 were designed using primer design software: PIEZO1-F, CCTGTCCGCCTACCAGATCCG; PIEZO1-R, ACCCATGCCGTACTTGACGAT. Relative gene expression was calculated using the 2-^ΔΔCt^ method.

### 2.6 Cell Culture and Treatment

Human bronchial epithelial cells (BEAS-2B, CRL-3588, American Type Culture Collection, USA) were cultured in an epithelial culture medium containing 10% fetal bovine serum at 37°C in a 95% humidified condition with 5% CO_2_. To determine the effect of Piezo1 on the expression of E-cadherin, α-SMA, and vimentin, cells were treated with the presence or absence of Yoda1 (10 μmol/L) or GsMTx4 (1 μmol/L GsMTx4). Piezo1, E-cadherin (1:200; ab231303, Abcam), α-SMA (1:300; 701457, Invitrogen), and vimentin (1:200; ab92547, Abcam) expressions were assessed by immunofluorescence and Western blot as described above.

### 2.7 Cell Transfection of siRNAs

To knock down YAP, cells (70 to 80% confluent) in six-well plates were incubated with a mixture of siRNA YAP (100 nM), Lipofectamine 3000, and Opti-MEM for 48 hours, followed by cell stimulation. Control cells were treated with the transfection reagent and scrambled siRNA. siRNA YAP was designed and obtained from RiboBio, China.

### 2.8 Nuclear and Cytoplasmic Protein Extraction

The nuclear and cytoplasmic protein fractions of the BEAS-2B cells were isolated using NEPER nuclear and cytoplasmic extraction reagents (ThermoFisher Scientific, USA) in accordance with the manufacturer’s instructions. Glyceraldehyde-3-phosphate dehydro-genase (GAPDH) or histone 3 (H3) was employed as a control for the cytoplasmic or nuclear protein fractions, respectively.

### 2.9 Calcium Imaging

BEAS-2B cells were incubated in Fluo-4 AM (5 μM; Beyotime, China) following the manufacturer’s instructions. Cells were then washed with Hank’s balanced salt solution and placed in 5% CO2 at 37°C for 10 minutes. Images were obtained using a Leica SP8 scanning microscope (Leica SDPTOP HS6).

### 2.10 Statistical Analysis

Each experiment was performed at least three times independently. All data were expressed as the mean ± standard deviation (SD). Statistical significance was determined using one-way ANOVA with Tukey’s HSD post-hoc test after confirming normality (Shapiro-Wilk) and homogeneity of variance (Levene’s test). Non-parametric data were analyzed *via* Kruskal-Wallis with Dunn’s correction. Statistical significance was set at *P < 0.05*. Statistical analysis was performed using GraphPad Prism 8.0 (CA, USA). All analyses were performed using SPSS software (version 22.0; IBM Corp.).

## 3 RESULTS

### 3.1 Increased Expression of Piezo1 and EMT in OB

To investigate the potential association between Piezo1 expression levels and OB progression, Piezo1 labeling was performed on trachea tissue after orthotopic trachea transplantation. HE and Masson’s trichrome staining showed a higher occlusion rate and fibrosis in specimens obtained from the allograft group than those from the syngraft group (Figs. **1A**-**C**). Increased expression of Piezo1 mRNA and protein levels in the tracheal tissue of the allograft group was observed by quantitative PCR, Western blot, and immunofluorescence assay as compared to those from the syngraft group (Figs. **1D**-**G**). Previous studies have demonstrated that enhanced EMT is related to the progression of OB; therefore, we further examined the protein expression of EMT markers in the trachea.

Western blot and immunofluorescence staining detected that the levels of EMT-related markers α-SMA and vimentin were up-regulated while the level of E-cadherin was decreased in the allograft group as compared with those from the syngraft group (Figs. **1H**-**J**). Together, the above findings demonstrated that OB was associated with high expression of Piezo1 and EMT in the allograft trachea.

### 3.2 Piezo1 Activation by Yoda1 Promotes EMT and OB

A Piezo1-specific agonist, Yoda1, was applied to investigate whether Piezo1 activation contributes to EMT and OB in mice. Compared to the syngraft group, stimulation with Yoda1 could promote the occlusion rate and fibrosis in the trachea of the allograft group (Figs. **2A**-**C**). We also examined the expression of EMT-related proteins in the trachea tissues, as shown in Fig. (**2D**-**F**). Yoda1 upregulated the expression of α-SMA and vimentin proteins and decreased E-cadherin protein expression compared with the allograft group, as determined by Western blot analysis and immunofluorescence staining. To further validate the effects of Piezo1 on EMT markers *in vitro,* we observed that treatment with Yoda1 resulted in the upregulation of α-SMA and vimentin and suppressed E-cadherin expression compared to the control group, as evidenced by Western blot analysis (Figs. **2H**-**I**). Additionally, immunofluorescence staining was conducted to further verify the changes in the levels of E-cadherin, α-SMA, and vimentin (Fig. **2G**), and the results were consistent with those obtained by Western blot. These data confirmed that Piezo1 activation plays an important role in EMT and OB progression.

### 3.3 Inhibition of Piezo1 Reduces EMT and Protects against OB

Given the positive association between Piezo1 and EMT described above, we further aimed to verify the effects of Piezo1 inhibition by GsMTx4, a Piezo1 channel antagonist, on EMT and OB progression. As shown in Fig. (**3A**-**C**), we observed that GsMTx4 application significantly inhibited airway occlusion and fibrotic deposition in the airways of the Allograft+ GsMTx4 group compared to the allograft group, as evidenced by HE and Masson’s Trichrome staining. Furthermore, Western blot analysis and immuno-fluorescence staining showed that the upregulation of α-SMA and vimentin protein and the decrease in E-cadherin protein expression in the allograft group were significantly repressed by Piezo1 inhibition with GsMTx4 (Figs. **3D**-**F**). Additionally, an *in vitro* experiment was conducted to further substantiate the changes in E-cadherin, α-SMA, and vimentin levels caused by Piezo1 inhibition with GsMTx4, and the results were consistent with those obtained by Western blot and immunofluorescence staining *in vivo* (Figs. **3G**-**I**). Notably, these results confirmed that Piezo1 inhibition could mitigate EMT and protect against OB.

### 3.4 Piezo1 Modulates EMT Processes through the YAP-Dependent Pathway

YAP, as a co-transcriptional activator, plays a critical role in EMT, and its signaling pathway can be regulated by mechanical stress. Since mechanical stress induces Piezo1 activation, we hypothesized that Piezo1 activates YAP to promote EMT. We observed that the allograft group showed greater YAP expression than the syngraft group, and Yoda1 could elevate the YAP expression level in both the syngraft and allograft groups. However, there is no significant difference between the syngraft and syngraft+Yoda1 groups. In addition, the results also showed that the p-YAP level in the allograft group was lower than the sygraft group, and decreased p-YAP was found in the allograft+ Yoda1 group compared to the allograft group (Fig. **4A** and **B**).

To further investigate the function of YAP in Piezo1-induced fibrosis and EMT, we pretreated the allograft group with a specific YAP inhibitor, verteporfin, followed by exposure to Yoda1. We found that inhibition of YAP attenuated the Yoda1-induced fibrosis and OB after allograft (Fig. **4C**-**E**). Western blots and immunofluorescence were performed to detect the protein expression levels of E-cadherin, α-SMA, and vimentin. As shown in Fig. (**4F**-**4H**), decreased expres-sion of α-SMA and vimentin and increased E-cadherin levels were observed in the allograft+ Yoda1+ verteporfin group as compared with the allograft+ Yoda1 group.

We further verified the role of YAP in the modulation of EMT by Piezo1 using YAP siRNA transfection in the BEAS-2B cells. Consistently, the expression of E-cadherin was increased, and α-SMA and vimentin were markedly lowered in the cells in the si-YAP group compared with the si-NC group. Yoda1 treatment induced a marked increase in mesenchymal marker expression, including α-SMA and vimentin over-expression, along with a decrease in the E-cadherin, which was reversed by si-YAP intervention in the si-YAP+Yoda1 group (Figs. **5A** and **B**). As demonstrated by immunofluorescence, si-YAP reversed Yoda1-induced EMT markers, including decreased expression of α-SMA and vimentin and increased E-cadherin levels (Fig. **5D**). These results indicated that YAP was involved in Piezo1-mediated EMT. Furthermore, nucleocytoplasmic separation analysis was performed to assess the subcellular localization of YAP. The result demonstrated that Yoda1 triggered YAP to translocate into the nucleus, whereas Piezo1 inhibition with GsMTx4 arrested YAP in the cytoplasm (Figs. **5C**, **E**, and **F**).

### 3.5 Piezo1 Drives YAP Activation by Regulating Ca^2+^ Level

Piezo1 has been shown to regulate Ca²⁺ homeo-stasis, with calcium acting as a second messenger involved in cellular processes such as contraction, signal transduction, gene transcription, and cell death. We hypothesized that Ca^2+^ is required for Piezo-induced YAP activation and EMT progression in OB. *In vitro*, we observed that Piezo1 activation by Yoda1 significantly increased Ca^2+^ fluorescence, whereas GsMTx4 reduced the Ca^2+^ fluorescence intensity (Fig. **6A**). To further verify the role of Ca^2+^ influx in Piezo-induced YAP activation, BEAS-2B cells were treated with or without 1,2-bis(2-aminophenoxy)ethane-N,N,N′,N′-tetraacetic acid (BAPTA)-AM, an intracellular Ca^2+^ chelator, following Yoda1 stimulation. The results demonstrated that BAPTA-AM intervention abolished the intracellular Ca^2+^ accumulation induced by Yoda1 (Fig. **6B**). As shown in the nucleocytoplasmic separation analysis, Yoda1 stimulation displayed increased YAP expression in the nucleus, while BAPTA-AM treatment made YAP largely localized in the cytoplasm (Figs. **6C**-**6E**). Additionally, BAPTA-AM intervention also reversed Yoda1-induced EMT marker expression, including down-regulated vimentin and α-SMA, along with increased E-cadherin expression (Figs. **6F** and **6G**). Consistently, BAPTA-AM reduced Yoda1-induced EMT markers as demonstrated by immunofluorescence staining, as shown in Fig. (**6H**). These findings indicated that intracellular Ca^2+^ accumulation is responsible for Piezo1-induced YAP nucleus translocation and EMT.

## 4 DISCUSSION

Piezo1 is a mechanosensitive ion channel that plays a crucial role in translating mechanical forces into biological signals. This function is critical to various physiological processes in the lung, including the regulation of pulmonary vascular contraction, alveolar dilation, and the maintenance of lung function [31-33]. It is predominantly expressed in non-excitable cells, such as endothelial cells and epithelial cells, where it translates respiratory mechanical stimuli into biochemical signals [34]. For instance, abnormal Piezo1 activation contributes to vascular remodeling and increased vascular resistance in primary or secondary pulmonary artery hypertension [35-37]. During lung injury repair, increased tissue extracellular matrix stiffness activates Piezo1 in fibroblasts, promoting their proliferation and extracellular matrix synthesis, thereby exacerbating fibrosis in multiple pulmonary fibrotic diseases, such as idiopathic pulmonary fibrosis, silicosis, or radiation-induced fibrosis, and connective tissue disease-associated interstitial lung disease [17, 38, 39]. Under mechanical stress, such as high vascular pressure, Piezo1 activation leads to the internalization and degradation of the endothelial adherent junction, resulting in severe pulmonary vascular permeability and edema [21, 40]. This mechanism is particularly associated with the pathological progression of ventilation-associated lung injury and acute respiratory distress syndrome [33, 41, 42]. Additionally, dysregulated Piezo1 activity could influence immune cell function to accelerate inflammatory response, potentially aggravating asthma and chronic obstructive pulmonary disease progression [43-45]. As per our knowledge, this is the first report about the role of Piezo1 in OB after lung transplant in mice. We speculated that Piezo1 overexpression may be driven by a synergy of mechanical stress from increased airway pressure and lung mechanics, fibrotic ECM stiffness, hypoxic conditions, TGF-β, and inflammatory cytokines, and positive feedback loops involving calcium and YAP/TAZ. Further studies involving the mechanism of Piezo1 expression in lung tissues from OB patients and the exploration of its regulatory pathways would provide more insights.

EMT plays a crucial role in the pathogenesis and remodeling of OB, a severe, irreversible complication involving small bronchioles in lung transplant recipients. The initial injury to the epithelial cells, often due to chronic immune responses, infections, or inhalation of toxic substances, triggers EMT [46, 47]. IL-17 can induce the overexpression of type V collagen, further promoting EMT and contributing to the fibrotic process in OB. A hallmark of this transition is the upregulation of mesenchymal markers such as α-SMA and vimentin, along with the downregulation of the epithelial marker E-cadherin. The mesenchymal cells derived from EMT contribute to the deposition of the extracellular matrix, leading to fibrosis and occlusion of the small bronchioles. This fibrotic process results in the obstruction of airflow and high airway pressure, causing significant mechanical stress in the small airway [7, 48]. At present, increased expression of Piezo1 and mesenchymal markers, such as vimentin and α-SMA, along with decreased epithelial marker E-cadherin level in the allograft trachea, suggests that OB is strongly associated with Piezo1 expression and EMT. Furthermore, Piezo1 activation with Yoda1 exacerbated EMT and occlusion rate, and Piezo1 inhibition with GsMTx4 reversed these effects, which confirmed that Piezo1 plays an important role in the EMT and OB progression. Some studies have reported that Piezo1 participates in the regulation of EMT in tissue development, wound healing, and cancer metastasis [49]. For instance, aberrant Piezo1 activation has been linked to enhanced EMT, contributing to tumor progression and metastasis in oral squamous cell carcinoma, hepatocellular carcinoma, and prostate cancer. Piezo1-mediated EMT enables cancer cells to detach from the primary tumor, invade surrounding tissues, and grow into secondary tumors [50-52]. In addition, Piezo1 exhibits essential roles in inducing cell survival, modulating immune cell activation, and enhancing angiogenesis to accelerate cancer metastasis [53, 54]. These findings also provide us with novel ideas for studying new mechanisms by which Piezo1 promotes OB progression.

Increased Piezo1 activity can influence the development of disease through a variety of signaling pathways, such as TGF-β/Smad, Wnt/β-catenin, PI3K/Akt, Rock pathway, MAPK/ERK, and Hippo/YAP pathway [55-57]. These mechanotransductive signalings orchestrate the cytoskeletal rearrangements and regulate downstream gene expression. YAP is the core transcription factor of the Hippo signaling cascade, which is essential for controlling organ development, tissue regeneration, and tumorigenesis [58, 59]. The interaction between Piezo1 and the YAP pathway is particularly significant in the context of mechanotransduction and cellular responses to mechanical stimuli. For instance, Piezo1 activation has been found to enhance osteogenic differentiation of aortic valve interstitial cells, which, in turn, modulates glutaminolysis and histone acetylation of RUNX2 promoters [60]. Activated Piezo1 can promote cell proliferation, survival, and migration, contributing to tumor growth and metastasis in hepatocellular carcinoma through the MAPK-YAP signaling pathway [61]. Wang *et* *al*. investigated the role of YAP1 and Piezo1 in the transformation of skin from photoaging to squamous cell carcinoma under UV radiation, which involves the dynamic changes of lymphatic vessels [62]. Han *et* *al*. reported that fluid shear stress-activated Piezo1 facilitates prostate cancer metastasis and enhances cancer cell motility [63]. Similarly, in fibrotic diseases, Piezo1 activation contributes to the pathological remodeling of tissues by promoting fibroblast activation and extracellular matrix secretion [64, 65]. Here, we demonstrated that YAP inhibitor verteporfin attenuated the Yoda1-induced EMT and fibrosis, indicating that the Piezo1-YAP-EMT signaling pathway plays a critical role in OB.

Previous research proved that Piezo1 activation results in elevated intracellular calcium levels, which trigger the MAPK signaling cascade, including the phosphorylation of JNK, p38, and ERK2. These kinases then facilitate the nuclear translocation of YAP, where it can regulate the expression of target genes involved in cell proliferation and survival [17, 66]. Piezo1 activation also enhances YAP nuclear translocation, leading to increased cell proliferation and reduced apoptosis in hepatocellular carcinoma [67]. Ca²⁺ influx, as a secondary messenger induced by Piezo1 activation, has been demonstrated to play a crucial role in various conditions, including athero-sclerosis, cardiac hypertrophy, endothelial inflam-mation, vascular remodeling, liver and pulmonary fibrosis, chronic obstructive pulmonary disease, and osteoarthritis [68, 69]. In this study, intracellular Ca^2+^ accumulation was increased by Piezo1 activation by Yoda1 as measured using Fluo-4 AM. Furthermore, BAPTA-AM, a Ca^2+^ influx antagonist, reduced Piezo1-mediated intracellular Ca^2+^ concentration and YAP translocation as well as the EMT process in OB. These findings indicated that Piezo1 could accelerate EMT and the occlusion rate of the graft *via* Ca^2+^ influx-dependent YAP activation in OB.

Piezo1 is activated by mechanical stimuli, such as stretch, shear stress, and pressure. This activation occurs in various cellular contexts, including endothelial cells, epithelial cells, and fibroblasts [70, 71]. Piezo1-induced Ca^2+^ influx also affects actin cytoskeleton dynamics that can alter the mechanical properties of the cell and influence the localization of YAP [72, 73]. Increased cytoskeletal tension generally promotes the nuclear localization of YAP, enhancing its transcriptional activity [22, 74]. One key pathway involves the Ca^2+^/calmodulin-dependent protein kinase II (CaMKII), which can phosphorylate and activate downstream effectors that modulate YAP activity. Activated Piezo1 leads to an influx of calcium ions (Ca^2+^), triggering downstream signaling to promote YAP translocation from the cytoplasm to the nucleus, modulates the expression of oncogenic genes, and accelerates tumor progression [75, 76]. Once in the nucleus, YAP can act as a transcriptional co-activator, regulating the expression of genes involved in EMT to accelerate OB.

The mechanical forces present in the lung, whether physiological or pathological, are translated into bio-chemical signals through the Piezo1 channel. Under-standing how these forces contribute to diseases, such as OB, could lead to the development of therapies that modulate mechanotransduction pathways. This may also shed light on the broader implications of mechano-transduction in lung pathology beyond OB, potentially impacting the treatment of other lung diseases.

## STUDY LIMITATIONS

Firstly, the specific molecular mechanisms by which Piezo1 regulates EMT and fibrosis require further investigation. This includes identifying the downstream effectors of Piezo1 signaling and understanding how they interact with other pathways implicated in OB. Secondly, the potential for combination therapies targeting Piezo1 alongside other fibrotic pathways should be explored to determine if a synergistic effect can be achieved. Lastly, the use of a murine orthotopic trachea transplantation model and BEAS-2B cell line culture system may not fully replicate the human OB condition. We aimed to illustrate the broader context of airway changes observed in larger airways that may accompany or influence the fibrotic alterations in the bronchioles. Future studies should include animal experiments using the orthotopic lung transplantation model and translational clinical trials to assess the efficacy of Piezo1 inhibitors in patients with OB.

## CONCLUSION

Our findings revealed that Piezo1 expression is upregulated in OB, and its activation induces the EMT process *via* the YAP-dependent pathway, suggesting a direct role in facilitating EMT and subsequent fibrotic remodeling (Fig. **7**). The present study provides novel insights into the pathological mechanisms underlying OB, implicating mechanical stress and mechano-sensitive ion channel Piezo1 as significant contributors to the OB progression by promoting EMT.

## Figures and Tables

**Fig. (1):**
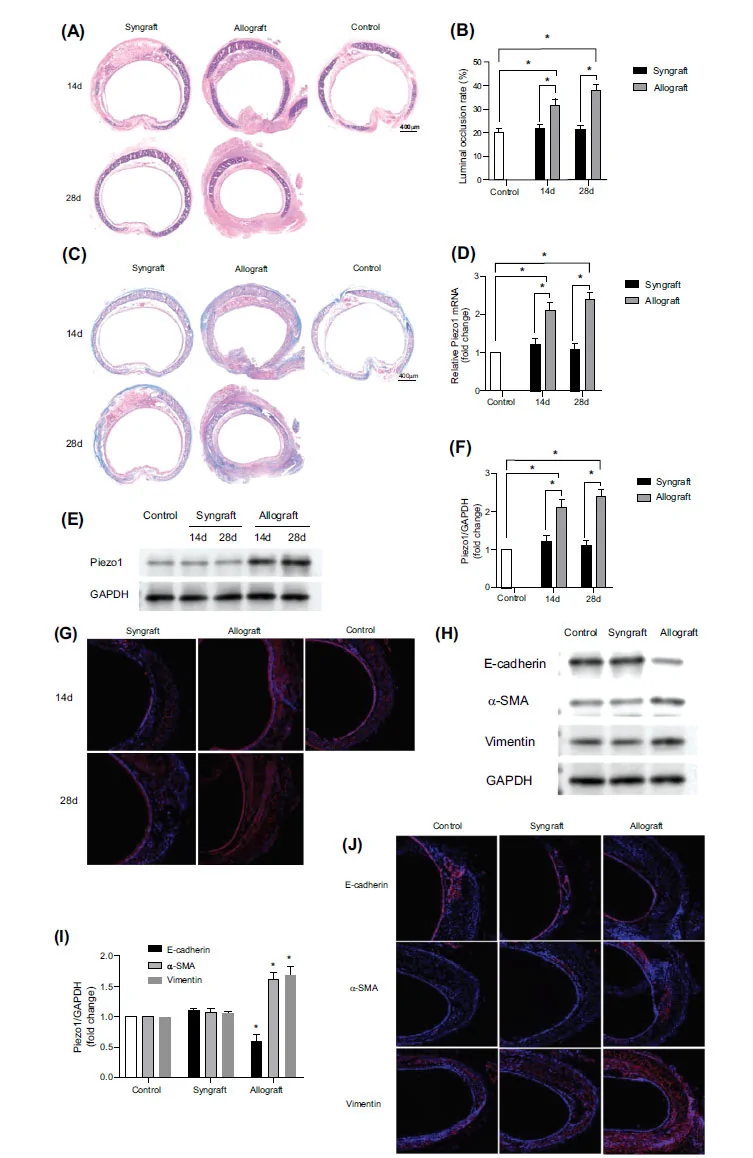
Increased expression of Piezo1 and EMT in OB *in vivo*. Graft tracheal samples were obtained from mice post-transplant. **(A, B)** The luminal occlusion rate was assessed in the HE-stained trachea slice (8×Magnification). **(C)** Collagen deposition within the tracheal epithelium was evaluated by Masson’s trichrome staining (8×Magnification). **(D)** Relative mRNA expression of Piezo1 by quantitative PCR assays. **(E, F)** Relative protein expression of Piezo1 in tracheal tissues by Western blot. **(G)** Representative fluorescence microscope images of Piezo1 staining (red) in the trachea (blue: nuclei, 10× Magnification). **(H, I)** EMT-related proteins were assayed by Western blot. **(J)** Representative fluorescence microscope images of EMT-related proteins in the trachea (red: E-cadherin, α-SMA, and vimentin, blue: nuclei). Data are expressed as mean ± SD, n=6, ^*^P<0.05.

**Fig. (2):**
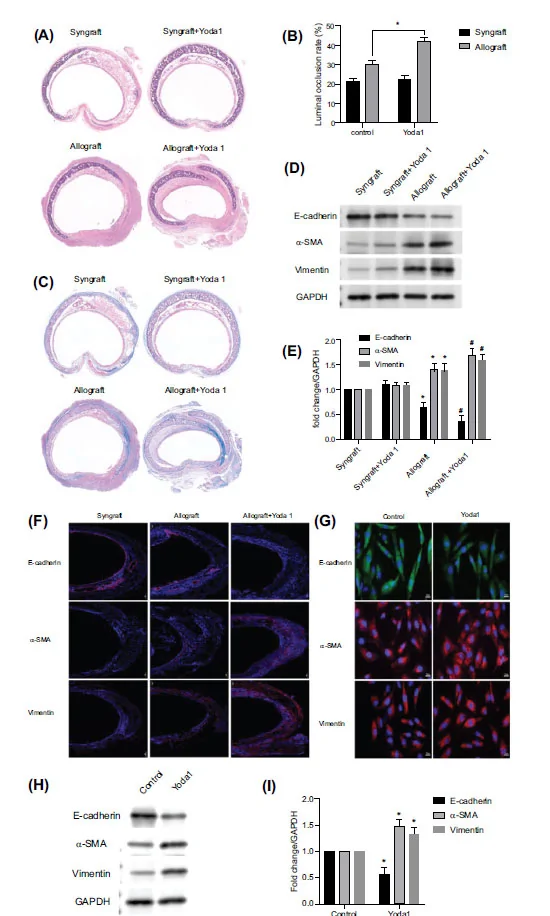
Piezo1 activation promotes EMT and OB *in vivo*. **(A, B)** The luminal occlusion rate was assessed in the HE-stained trachea slices (8× Magnification). **(C)** Collagen deposition within the tracheal epithelium was evaluated by Masson’s trichrome staining (8× Magnification). **(D, E)** EMT-related proteins in the trachea were assayed by Western blot. **(F)** Representative fluorescence microscope images of EMT-related proteins in trachea slices (red: E-cadherin, α-SMA, and vimentin, blue: nuclei, 10× Magnification). **(G)** Representative fluorescence microscope images of EMT-related proteins in BEAS-2B cells (green: E-cadherin, red: α-SMA and vimentin, blue: nuclei, scale bar: 20 μm). **(H** and **I)** EMT-related proteins in BEAS-2B cells were assayed by Western blot. Data are expressed as mean ± SD, n=6, ^*^P<0.05, ^#^P<0.05.

**Fig. (3):**
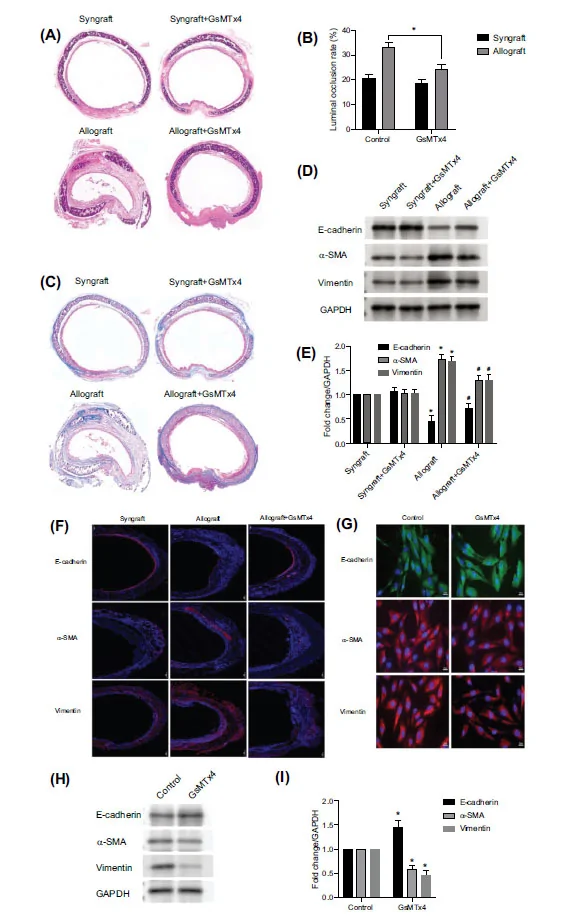
Inhibition of Piezo1 mitigates EMT and protects against OB *in vivo*. **(A, B)** The luminal occlusion rate was assessed in the HE-stained trachea slices (8× Magnification). **(C)** Collagen deposition within the tracheal epithelium was evaluated by Masson’s trichrome staining (8× Magnification). **(D, E)** EMT-related proteins in the trachea were assayed by Western blot. **(F)** Representative fluorescence microscope images of EMT-related proteins in the trachea (red: E-cadherin, α-SMA, and vimentin, blue: nuclei, 10× Magnification). **(G)** Representative fluorescence microscope images of EMT-related proteins in BEAS-2B cells (green: E-cadherin, red: α-SMA and vimentin, blue: nuclei, scale bar: 20 μm). **(H, I)** EMT-related proteins in BEAS-2B cells were assayed by Western blot. Data are expressed as mean ± SD, n=6, ^*^P<0.05, ^#^P<0.05.

**Fig. (4):**
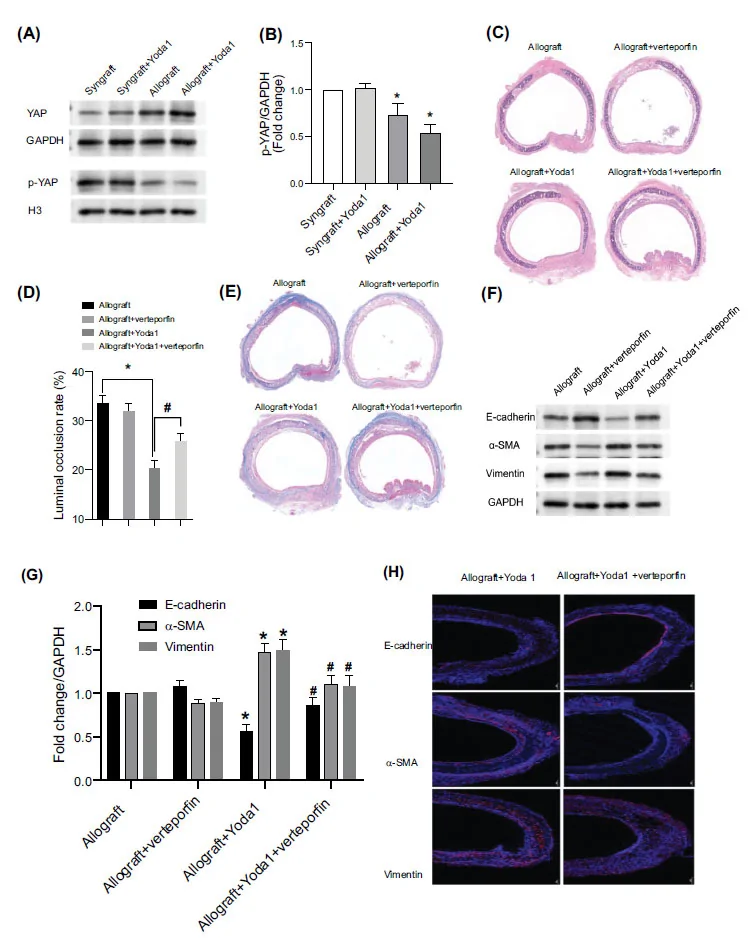
Piezo1 modulates EMT processes through the YAP-dependent pathway *in vivo*. **(A-B)** Representative immunoblots and analysis showed YAP and its phosphorylation expression in trachea tissues under Yoda1 stimulation. **(C, D)** The luminal occlusion rate was assessed in the HE-stained trachea slices (8× Magnification). **(E)** Collagen deposition within the tracheal epithelium was evaluated by Masson’s trichrome staining (8× Magnification). **(F, G)** EMT-related proteins in the trachea were assessed by Western blot. **(H)** Representative fluorescence microscope images of EMT-related proteins in the trachea (10× Magnification). Data are expressed as mean ± SD, n=6, ^*^P<0.05, ^#^P<0.05.

**Fig. (5):**
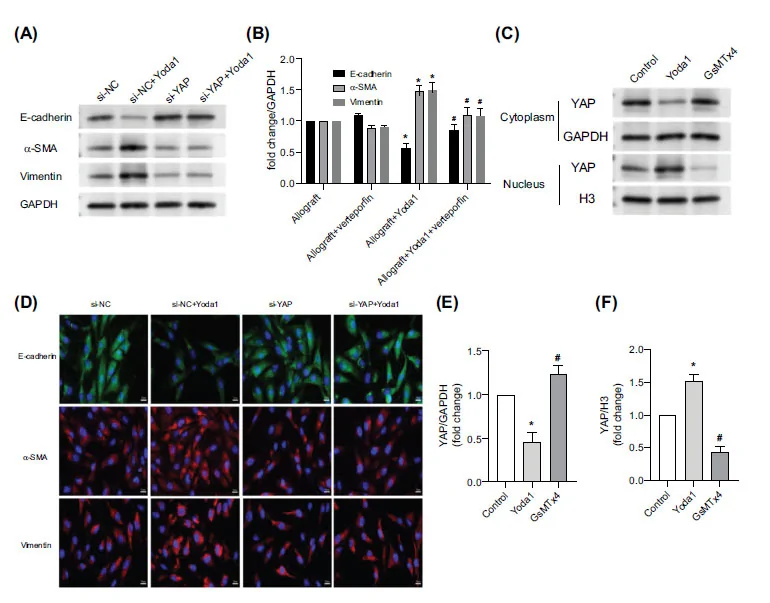
Piezo1 modulates EMT processes through the YAP-dependent pathway *in vitro*. **(A-B)** Representative immunoblots and analysis showed that knockdown of YAP reduced Yoda1-induced EMT markers *in vitro*. **(C)** Representative immunoblots showed that YAP induced nuclear translocation of Yoda1, whereas GsMTx4 inhibited this effect. **(D)** Representative fluorescence microscope images of EMT-related proteins showed that the knockdown of YAP reduced Yoda1-induced EMT *in vitro* (scale bar: 20 μm). **(E, F)** Densitometric data analysis showed that YAP nucleus translocation was induced by Yoda1 while inhibited by GsMTx4. Data are expressed as mean ± SD, n=6, ^*^P<0.05, ^#^P<0.05.

**Fig. (6):**
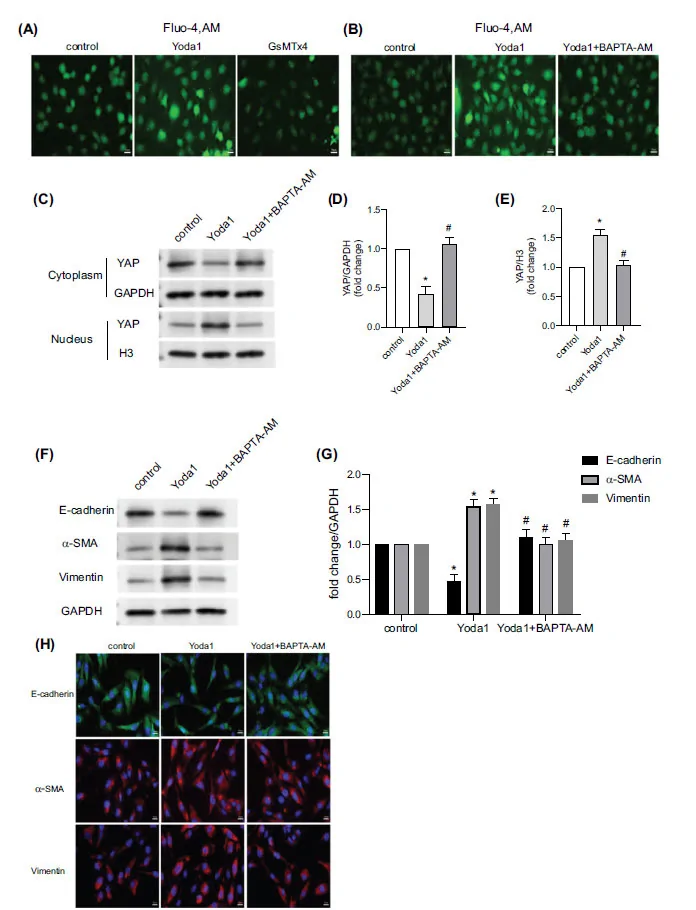
Piezo1 drives YAP activation by regulating Ca^2+^ influx in BEAS-2B cells. **(A)** Representative fluorescence imaging of intracellular Ca^2+^ was induced by Yoda1 while inhibited by GsMTx4 *in vitro* using Fluo-4 AM (scale bar: 20 μm). **(B)** BAPTA-AM intervention abolished the intracellular Ca^2+^ accumulation induced by Yoda1 *in vitro*. Representative fluorescence imaging of intracellular Ca^2+^ *in vitro*. **(C-E)** Western blot and data analysis showed that BAPTA-AM abolished Yoda1-induced YAP nucleus translocation. **(F, G)** Western blot and data analysis showed that BAPTA-AM repressed Yoda1-induced EMT *in vitro*. **(H)** Representative fluorescence microscope images of EMT-related proteins showed that BAPTA-AM repressed Yoda1-induced EMT (scale bar: 20 μm). Data are expressed as mean ±SD, n=6, ^*^P<0.05, ^#^P<0.05.

**Fig. (7):**
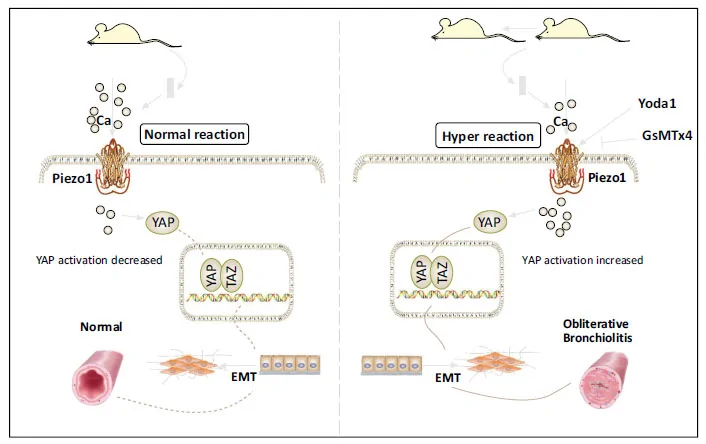
Schematic illustration of Piezo1-YAP signaling pathway mediated EMT contributing to the pathogenesis of OB.

## Data Availability

The datasets generated and/or analyzed during the current study are available from the corresponding author upon reasonable request.

## LIST OF ABBREVIATIONS

H&E: Hematoxylin and Eosin
YAP: Yes-associated protein
EMT: Epithelial-to-mesenchymal transition
OB: Obliterative bronchiolitis

## References

[1] Barker A.F., Bergeron A., Rom W.N., Hertz M.I. (2014). Obliterative bronchiolitis.. N Engl J Med.

[2] Andersson-Sjöland A., Erjefält J.S., Bjermer L., Eriksson L., Westergren-Thorsson G. (2009). Fibrocytes are associated with vascular and parenchymal remodelling in patients with obliterative bronchiolitis.. Respir Res.

[3] Willis B.C., Borok Z. (2009). Epithelial-mesenchymal transition: Potential role in obliterative bronchiolitis?. Thorax.

[4] Sato M., Keshavjee S., Liu M. (2009). Translational research: Animal models of obliterative bronchiolitis after lung transplantation.. Am J Transplant.

[5] Bellon H., Vandermeulen E., Verleden S.E. (2017). The effect of immunosuppression on airway integrity.. Transplantation.

[6] Andersson-Sjöland A., Thiman L., Nihlberg K. (2011). Fibroblast phenotypes and their activity are changed in the wound healing process after lung transplantation.. J Heart Lung Transplant.

[7] Vittal R., Fan L., Greenspan D.S. (2013). IL-17 induces type V collagen overexpression and EMT *via* TGF-β-dependent pathways in obliterative bronchiolitis.. Am J Physiol Lung Cell Mol Physiol.

[8] Borthwick L.A., Parker S.M., Brougham K.A. (2009). Epithelial to mesenchymal transition (EMT) and airway remodelling after human lung transplantation.. Thorax.

[9] Konoeda C., Koinuma D., Morishita Y. (2013). Epithelial to mesenchymal transition in murine tracheal allotrans-plantation: An immunohistochemical observation.. Transplant Proc.

[10] Fröse J., Chen M.B., Hebron K.E. (2018). Epithelial-mesenchymal transition induces podocalyxin to promote extravasation *via* ezrin signaling.. Cell Rep.

[11] Borthwick L.A., McIlroy E.I., Gorowiec M.R. (2010). Inflammation and epithelial to mesenchymal transition in lung transplant recipients: Role in dysregulated epithelial wound repair.. Am J Transplant.

[12] Xiao B. (2024). Mechanisms of mechanotransduction and physiological roles of PIEZO channels.. Nat Rev Mol Cell Biol.

[13] Wang Y., Wang J., Zhang J. (2024). Stiffness sensing *via* Piezo1 enhances macrophage efferocytosis and promotes the resolution of liver fibrosis.. Sci Adv.

[14] Zhang J., Li J., Hou Y. (2024). Osr2 functions as a biomechanical checkpoint to aggravate CD8+ T cell exhaustion in tumor.. Cell.

[15] Zhong G., Su S., Li J. (2023). Activation of Piezo1 promotes osteogenic differentiation of aortic valve interstitial cell through YAP-dependent glutaminolysis.. Sci Adv.

[16] Bagley D.C., Russell T., Ortiz-Zapater E. (2024). Broncho-constriction damages airway epithelia by crowding-induced excess cell extrusion.. Science.

[17] Zheng M., Yao Y., Borkar N.A. (2024). Piezo channels modulate human lung fibroblast function.. Am J Physiol Lung Cell Mol Physiol.

[18] Zhong M., Wu W., Kang H. (2020). Alveolar stretch activation of endothelial piezo1 protects adherens junctions and lung vascular barrier.. Am J Respir Cell Mol Biol.

[19] Hurrell B.P., Shen S., Li X. (2024). Piezo1 channels restrain ILC2s and regulate the development of airway hyperreactivity.. J Exp Med.

[20] Luo M., Gu R., Wang C. (2024). High stretch associated with mechanical ventilation promotes piezo1-mediated migration of airway smooth muscle cells.. Int J Mol Sci.

[21] Friedrich E.E., Hong Z., Xiong S. (2019). Endothelial cell Piezo1 mediates pressure-induced lung vascular hyperpermeability *via* disruption of adherens junctions.. Proc Natl Acad Sci USA.

[22] Koo J.H., Guan K.L. (2018). Interplay between YAP/TAZ and Metabolism.. Cell Metab.

[23] Lange A.W., Sridharan A., Xu Y., Stripp B.R., Perl A.K., Whitsett J.A. (2015). Hippo/Yap signaling controls epithelial progenitor cell proliferation and differentiation in the embryonic and adult lung.. J Mol Cell Biol.

[24] Aharonov A., Shakked A., Umansky K.B. (2020). ERBB2 drives YAP activation and EMT-like processes during cardiac regeneration.. Nat Cell Biol.

[25] Malgundkar S.H., Burney I., Al Moundhri M. (2020). FAT4 silencing promotes epithelial-to-mesenchymal transition and invasion *via* regulation of YAP and β-catenin activity in ovarian cancer.. BMC Cancer.

[26] Gao C., Quan M.Y., Chen Q.J. (2021). Yap1-2 isoform is the primary mediator in TGF-β1 induced EMT in pancreatic cancer.. Front Oncol.

[27] Zeng S.H.G., Xie J.H., Zeng Q.Y. (2021). lncRNA PVT1 promotes metastasis of non-small cell lung cancer through EZH2-mediated activation of Hippo/NOTCH1 signaling pathways.. Cell J.

[28] Wang C., Xia T., Jiang K. (2016). Apoptosis of the tracheal epithelium can increase the number of recipient bone marrow–derived myofibroblasts in allografts and exacerbate obliterative bronchiolitis after tracheal transplantation in mice.. Transplantation.

[29] Wang J., Ma Y., Sachs F., Li J., Suchyna T.M. (2016). GsMTx4-D is a cardioprotectant against myocardial infarction during ischemia and reperfusion.. J Mol Cell Cardiol.

[30] Liu S., Pan X., Cheng W. (2020). Tubeimoside I antagonizes yoda1-evoked piezo1 channel activation.. Front Pharmacol.

[31] Yao Y., Zheng M., Borkar N.A. (2024). Role of STIM1 in stretch-induced signaling in human airway smooth muscle.. Am J Physiol Lung Cell Mol Physiol.

[32] Zhang X., Liu J., Deng X., Bo L. (2024). Understanding COVID-19-associated endothelial dysfunction: Role of PIEZO1 as a potential therapeutic target.. Front Immunol.

[33] Fang X.Z., Li M., Wang Y.X. (2023). Mechanosensitive ion channel Piezo1 mediates mechanical ventilation-exacerbated ARDS-associated pulmonary fibrosis.. J Adv Res.

[34] Liang G.P., Xu J., Cao L. (2019). Piezo1 induced apoptosis of type II pneumocytes during ARDS.. Respir Res.

[35] Wang Z., Chen J., Babicheva A. (2021). Endothelial upregulation of mechanosensitive channel Piezo1 in pulmonary hypertension.. Am J Physiol Cell Physiol.

[36] Chen J., Rodriguez M., Miao J. (2022). Mechanosensitive channel Piezo1 is required for pulmonary artery smooth muscle cell proliferation.. Am J Physiol Lung Cell Mol Physiol.

[37] Porto Ribeiro T., Barbeau S., Baudrimont I. (2022). Piezo1 channel activation reverses pulmonary artery vasocons-triction in an early rat model of pulmonary hypertension: The role of Ca^2+^ influx and Akt-eNOS pathway.. Cells.

[38] Freeberg M.A.T., Perelas A., Rebman J.K., Phipps R.P., Thatcher T.H., Sime P.J. (2021). Mechanical feed-forward loops contribute to idiopathic pulmonary fibrosis.. Am J Pathol.

[39] Huang J.Q., Zhang H., Guo X.W. (2021). Mechanically activated calcium channel PIEZO1 modulates radiation-induced epithelial-mesenchymal transition by forming a positive feedback with TGF-β1.. Front Mol Biosci.

[40] Diem K., Fauler M., Fois G. (2020). Mechanical stretch activates piezo1 in caveolae of alveolar type I cells to trigger ATP release and paracrine stimulation of surfactant secretion from alveolar type II cells.. FASEB J.

[41] Jia Q., Yang Y., Chen X., Yao S., Hu Z. (2022). Emerging roles of mechanosensitive ion channels in acute lung injury/acute respiratory distress syndrome.. Respir Res.

[42] Jiang L., Zhang Y., Lu D. (2021). Mechanosensitive Piezo1 channel activation promotes ventilator-induced lung injury *via* disruption of endothelial junctions in ARDS rats.. Biochem Biophys Res Commun.

[43] Zhou J., Zhou X., Xu R. (2021). The degradation of airway epithelial tight junctions in asthma under high airway pressure is probably mediated by Piezo-1.. Front Physiol.

[44] Kelley B., Zhang E.Y., Khalfaoui L. (2023). Piezo channels in stretch effects on developing human airway smooth muscle.. Am J Physiol Lung Cell Mol Physiol.

[45] Aranda L.C., Ribeiro I.C., Freitas T.O. (2023). Enhanced respiratory frequency response to lower limb mechanore-ceptors activation in patients with chronic obstructive pulmonary disease.. Med Sci Sports Exerc.

[46] Renaud-Picard B., Vallière K., Toussaint J. (2020). Epithelial-mesenchymal transition and membrane microparticles: Potential implications for bronchiolitis obliterans syndrome after lung transplantation.. Transpl Immunol.

[47] Hodge S., Holmes M., Banerjee B. (2009). Posttransplant bronchiolitis obliterans syndrome is associated with bronchial epithelial to mesenchymal transition.. Am J Transplant.

[48] Wang J., He Y., Yang G., Li N., Li M., Zhang M. (2020). Transient receptor potential canonical 1 channel mediates the mechanical stress-induced epithelial-mesenchymal transition of human bronchial epithelial (16HBE) cells.. Int J Mol Med.

[49] He J., Shan S., Li Q., Fang B., Xie Y. (2022). Mechanical stretch triggers epithelial-mesenchymal transition in keratinocytes through piezo1 channel.. Front Physiol.

[50] Bo H., Wu Q., Zhu C., Zheng Y., Cheng G., Cui L. (2024). PIEZO1 acts as a cancer suppressor by regulating the ROS / Wnt /β‐catenin axis.. Thorac Cancer.

[51] Lopez-Cavestany M., Hahn S.B., Hope J.M. (2023). Matrix stiffness induces epithelial-to-mesenchymal transition *via* Piezo1-regulated calcium flux in prostate cancer cells.. iScience.

[52] Li Y., Xu C., Sun B., Zhong F., Cao M., Yang L. (2022). Piezo1 promoted hepatocellular carcinoma progression and EMT through activating TGF-β signaling by recruiting Rab5c.. Cancer Cell Int.

[53] Ezzo M., Spindler K., Wang J.B. (2024). Acute contact with profibrotic macrophages mechanically activates fibroblasts *via* αvβ3 integrin–mediated engagement of Piezo1.. Sci Adv.

[54] Aykut B., Chen R., Kim J.I. (2020). Targeting Piezo1 unleashes innate immunity against cancer and infectious disease.. Sci Immunol.

[55] Feng S., Yu Z., Yang Y., Xiong Q., Yan X., Bi Y. (2024). Mechanosensitive Piezo1 channels promote neurogenic bladder fibrosis *via* regulating TGF-β1/smad and Hippo/YAP1 pathways.. Exp Cell Res.

[56] He X., Liu Y., Dai Z. (2024). Yoda1 pretreated BMSC derived exosomes accelerate osteogenesis by activating phospho-ErK signaling *via* Yoda1-mediated signal transmission.. J Nanobiotechnology.

[57] Zhan H., Xie D., Yan Z. (2024). Fluid shear stress-mediated Piezo1 alleviates osteocyte apoptosis by activating the PI3K/Akt pathway.. Biochem Biophys Res Commun.

[58] Zhong Z., Jiao Z., Yu F.X. (2024). The Hippo signaling pathway in development and regeneration.. Cell Rep.

[59] Moya I.M., Halder G. (2019). Hippo–YAP/TAZ signalling in organ regeneration and regenerative medicine.. Nat Rev Mol Cell Biol.

[60] Gan D., Tao C., Jin X. (2024). Piezo1 activation accelerates osteoarthritis progression and the targeted therapy effect of artemisinin.. J Adv Res.

[61] Liu S., Xu X., Fang Z. (2021). Piezo1 impairs hepatocellular tumor growth *via* deregulation of the MAPK-mediated YAP signaling pathway.. Cell Calcium.

[62] Yang Y.L., Zhou C., Chen Q. (2023). YAP1/Piezo1 involve in the dynamic changes of lymphatic vessels in UVR-induced photoaging progress to squamous cell carcinoma.. J Transl Med.

[63] Han Y., Liu C., Zhang D. (2019). Mechanosensitive ion channel Piezo1 promotes prostate cancer development through the activation of the Akt/mTOR pathway and acceleration of cell cycle.. Int J Oncol.

[64] Fu Y., Wan P., Zhang J. (2021). Targeting mechanosensitive piezo1 alleviated renal fibrosis through p38MAPK-YAP pathway.. Front Cell Dev Biol.

[65] Xu H.Q., Guo Z.X., Yan J.F. (2023). Fibrotic matrix induces mesenchymal transformation of epithelial cells in oral submucous fibrosis.. Am J Pathol.

[66] Dombroski J.A., Hope J.M., Sarna N.S., King M.R. (2021). Channeling the force: Piezo1 mechanotransduction in cancer metastasis.. Cells.

[67] Li M., Zhang X., Wang M. (2022). Activation of Piezo1 contributes to matrix stiffness‐induced angiogenesis in hepatocellular carcinoma.. Cancer Commun.

[68] Thiel G., Schmidt T., Rössler O.G. (2021). Ca^2+^ microdomains, calcineurin and the regulation of gene transcription.. Cells.

[69] Man K.N.M., Bartels P., Horne M.C., Hell J.W. (2020). Tissue-specific adrenergic regulation of the L-type Ca 2+ channel Ca V 1.2.. Sci Signal.

[70] Tian C., Lyu T., Zhao X., Wang R., Wu Y., Yang D. (2024). Piezo1 channel: A global bibliometric analysis from 2010 to 2024.. Channels.

[71] Kinsella J.A., Debant M., Parsonage G. (2024). Pharmacology of PIEZO1 channels.. Br J Pharmacol.

[72] Morachevskaya E.A., Sudarikova A.V. (2021). Actin dynamics as critical ion channel regulator: ENaC and Piezo in focus.. Am J Physiol Cell Physiol.

[73] Nourse J.L., Pathak M.M. (2017). How cells channel their stress: Interplay between Piezo1 and the cytoskeleton.. Semin Cell Dev Biol.

[74] Hooglugt A., van der Stoel M.M., Shapeti A. (2024). DLC1 promotes mechanotransductive feedback for YAP *via* RhoGAP-mediated focal adhesion turnover.. J Cell Sci.

[75] Lan Y., Lu J., Zhang S. (2024). Piezo1‐mediated mechano-transduction contributes to disturbed flow‐induced atherosclerotic endothelial inflammation.. J Am Heart Assoc.

[76] Yun J., Hansen S., Morris O. (2023). Senescent cells perturb intestinal stem cell differentiation through Ptk7 induced noncanonical Wnt and YAP signaling.. Nat Commun.

